# The quest for the “HOIL-1” grail of T-cell development

**DOI:** 10.1038/s41418-021-00856-2

**Published:** 2021-08-31

**Authors:** Nuno L. Alves, Pedro Ferreirinha

**Affiliations:** 1 i3S—Instituto de Investigação e Inovação em Saúde, Universidade do Porto, Porto, Portugal; 2 IBMC—Instituto de Biologia Molecular e Celular, Universidade do Porto, Porto, Portugal

**Keywords:** Signal transduction, Lymphocytes

Given the current interest in the design of new T cell-driven immunotherapy, it is crucial to understand the principles that control T-cell development. As the generative organ of functionally diverse and self-tolerant T cells, the thymus is central for the establishment of immunity against cancer and pathogens and prevention of autoimmunity. Within the thymus, cortical (c) and medullary (m) thymic epithelial cells (TECs) play an essential role in T-cell development. While cTECs mediate T-cell lineage commitment and positive selection, mTECs regulate negative selection and T-regulatory cell differentiation. The capacity of mTECs to imposed self-tolerance is conferred by their capacity to promiscuously expressed tissue-restricted antigens, a process that is in part regulated by Aire and Fezf2. As a result, defects in TECs can lead to immunodeficiency or autoimmunity [1]. Thus, the identification of new regulators of TEC biology is fundamental to understand the foundations of immunity and develop new therapeutics to correct dysfunctional T-cell responses.

In this issue of Cell Death and Differentiation, Jain et al. demonstrate that LUBAC (Linear Ubiquitin Chain Assembly Complex) is required for the maintenance of TEC microenvironments, and hence to sustain thymic function [2]. LUBAC is a heterotrimeric E3 ligase complex composed of HOIL-1, HOIP and SHARPIN. The complex is capable of linear ubiquitination members of the canonical nuclear factor kappa B (NF-κB) pathway, thereby regulating TNF receptor (TNFR) signaling [3]. Given the well-established role of NF-κB pathway in TEC differentiation, in particularly in mTECs [4], it is logical to interrogate the individual regulatory role of the distinct components of LUBAC in TEC homeostasis. To do so, the authors employed either mice with TEC-specific deletion of *Hoil-1* and *Hoip* genes (HOIL-1 and HOIP cKO) or mice with a spontaneous germline mutation in the *Sharpin* gene (SHARPIN^cpdm^). They started by determining the TEC-intrinsic role for LUBAC in thymopoisesis in adult mice. Their first important finding was that deficiency in HOIL-1 and HOIP led to a severe thymic atrophy, which extended to a reduction in the number naive T cells [2]. The peripheral T-cell lymphopenia in cKO mice highlights the requirements of a regular thymic function to maintain a normal T-cell homeostasis [5]. Future studies may consider to examine the TCR repertoire of T cells that arise from mutant thymus and their capacity to mount protective immune responses. The severe phenotypes caused by HOIL-1 and HOIP-deficiency contrasted with a moderate impact in SHARPIN^cpdm^ mice. Albeit the confounding effects of broad SHARPIN-deficiency in SHARPIN^cpdm^ mice preclude the assessment of the specific function of SHARPIN in TECs [3], this study indicates that HOIL-1 and HOIP are essential in TECs to sustain thymic function in young and adult mice.

The differentiation of cTECs and mTECs is a dynamic process that starts during early embryogenesis and continues throughout life, with the prototypical cortical-medullary compartmentalization achieved in the adulthood [6]. Further analysis on the cell-autonomous role of HOIL-1 and HOIP in TEC differentiation in the corresponding cKO revealed a marked reduction in the number of mTECs, including mTEC^lo^, mTEC^hi^ and Aire^+^ cells. The loss in mTECs appeared in the embryo and perpetuated during postnatal life. Yet, the aforementioned mTEC subsets developed in the absence of HOIL-1 and HOIP [2], suggesting that LUBAC guides the expansion and maintenance of mTEC subsets, but is dispensable for their differentiation. Although the establishment of the murine mTEC compartment is initiated in the embryo, it must be maintained through life. Particularly, mature Aire^+^mTECs turnover approximately every 7–10 days [7], implicating a regular replacement by their upstream precursors. Recent studies revealed a plethora of new dedicated mTEC precursors and how they contribute to the maintenance of mTEC niches [7]. A future in-depth analysis on the generation and maintenance of mTEC precursors in cKO mice may inform on specific development checkpoints controlled by HOIL-1 and HOIP. In contrast to mTECs, cTECs become mostly affected only in the adult thymus. As a result, the cortical-medullary regions of HOIL-1 and HOIP cKO thymi were severely disrupted. Together, their findings suggest that HOIL-1 and HOIP are relevant for mTEC survival and growth throughout life and cTEC maintenance in the adult period.

Given that the deficiency in HOIL-1 phenocopies the thymic failures caused by the loss of HOIP, the authors focused on the first to examine the underlining molecular basis for the role of LUBAC in TECs. Transcriptional analysis of TEC subsets at 2 weeks of age, a time point that precedes the development of a severe thymic phenotype, revealed thousands of differentially expressed genes (DEG) in HOIL-1-deficient cTEC (~3000) and mTEC^hi^ (~5700). Gene ontology analysis pointed to changes in cell adhesion, projection and morphology in cKO cTECs, and alterations in cell cycle and metabolism in mTEC^hi^ counterparts [2]. These findings support that LUBAC-dependent signaling control a broad transcriptional program in TECs associated to basic processes in cell biology. It remains undetermined whether DEG include tissue-restricted antigen and direct or indirect NF-κB targets. These future analyses would enable to evaluate the extend of abnormal NF-κB activation in cKO TECs. Additionally, further studies may clarify whether the structural and transcriptional changes found in cKO cTECs are cell-autonomous, and directly induced by HOIL-1- and HOIP-deficiency. Alternatively, mTEC alterations might perturb the organization and function of cTECs, implicating a complex interplay between TEC microenvironments.

The fact that LUBAC-mediated signaling prevents caspase-8-dependent apoptosis and/or by MLKL-dependent necroptosis [8], led the authors to consider that the loss of TECs in HOIL-1 cKO was primarily a consequence of cell death. Indeed, HOIL-1 deficiency led to an increase in the expression of genes involved in receptor-mediated programmed cell death. Mechanistically, the study elegantly showed that combined deficiency in caspase-8 and MLKL rescues the defects in thymocyte and peripheral T cells induced by HOIL-1 deficiency, but only moderately correct the numbers and distribution of mTECs [2]. These findings implicate that LUBAC regulates other pathways supporting the maintenance and growth of mTECs. One candidate put forward in the study is the p53-mediated apoptotic pathway. Albeit mTECs require basal levels of p53 to maintain their functionality [9], its upregulation might induce the transcription of many genes involved in cell cycle arrest, DNA repair and apoptosis [10]. Hence, one prediction would be that HOIL-1-deficient TECs present an elevated expression of p53 targets.

The differentiation of mTECs depends on the cooperative action of members of TNFR superfamily (TNFRSF), including receptor activator of NF-κB (RANK), CD40 and lymphotoxin β receptor (LTβR), leading to the activation of NF-κB-dependent maturation program. The reduced mTEC microenvironment found in HOIL-1 and HOIP cKO is reminiscent of the alterations provoked by deficiency in members of the TNFRSF, particularly RANK or/and CD40, or those with loss of the NF-κB signaling proteins [4]. Disruption of RANK, CD40 and LTβR or their downstream signaling components, such as TRAF6, NIK and RelB, results in the development of organ-specific autoimmunity [4]. As such, the reported loss of mTECs and T-regulatory cells in HOIL-1 and HOIP cKO thymus may be equally predictive of defects in self-tolerance and development of autoimmunity. Future studies will be necessary to elucidate the role of LUBAC in negative selection and T-regulatory cell function, and how this expands to the establishment of peripheral T-cell tolerance. Moreover, it remains unclear whether the function of LUBAC in TECs associates with the well-established role of TNFR signaling in mTECs. One possibility is that LUBAC controls NF-κB signaling downstream of RANK, CD40, and LTβR, fine-tuning the responsiveness of mTECs, and their precursors, to these signals. An alternative, and perhaps more speculative, scenario is that LUBAC regulates the activation of the canonical NF-κB pathway engaged by other TNFRSF members, which would connect inflammatory signals to cell death.

Together, the discoveries by Jain et al. extend beyond our knowledge how TEC perceive and integrate critical signals into molecular programs that drive their survival, differentiation and function. It will be fascinating to continue following how LUBAC contributes to thymic biology and T-cell immunity. Knowledge in this area will also inform on novel therapeutic approaches to repair a dysfunctional thymus Fig. 1.

**Fig. 1:**
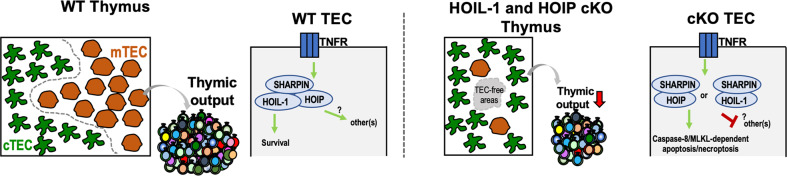
Cortical (c) and medullary (m) thymic epithelial cell (TEC) compartments in WT and HOIL-1 and HOIP cKO mice. TEC-specific deletion of components of LUBAC E3 ligase complex provoke a deterioration of TEC microenvironment and significantly reduced T-cell production by the thymus. In WT TECs (left), integral LUBAC-mediated signaling induces cell survival and regulates other yet to identified pathways important for TEC biology. In cKO TECs (right), attenuated LUBAC-mediated signaling leads to apoptosis/necroptosis and deregulate other routes important for TEC biology.

## References

[1] Abramson J, Anderson G (2017). Thymic epithelial cells. Annu Rev Immunol.

[2] Jain R, Zhao K, Sheridan JM, Heinlein M, Kupresanin F, Abeysekera W, et al. Dual roles for LUBAC signaling in thymic epithelial cell development and survival. Cell Death Differ. 2021. 10.1038/s41418-021-00850-8. [Epub ahead of print]. 10.1038/s41418-021-00850-8 PMC8481470 34381167

[3] Fiil BK, Gyrd-Hansen M (2021). The Met1-linked ubiquitin machinery in inflammation and infection. Cell Death Differ.

[4] Irla M, Hollander G, Reith W (2010). Control of central self-tolerance induction by autoreactive CD4+ thymocytes. Trends Immunol.

[5] Almeida AR, Rocha B, Freitas AA, Tanchot C (2005). Homeostasis of T cell numbers: from thymus production to peripheral compartmentalization and the indexation of regulatory T cells. Semin Immunol.

[6] Anderson G, Takahama Y (2012). Thymic epithelial cells: working class heroes for T cell development and repertoire selection. Trends Immunol.

[7] Alves NL, Ribeiro AR (2016). Thymus medulla under construction: time and space oddities. Eur J Immunol.

[8] Verboom L, Hoste E, van Loo G (2021). OTULIN in NF-kappaB signaling, cell death, and disease. Trends Immunol.

[9] Rodrigues PM, Ribeiro AR, Perrod C, Landry JJM, Araujo L, Pereira-Castro I (2017). Thymic epithelial cells require p53 to support their long-term function in thymopoiesis in mice. Blood..

[10] Vousden KH, Prives C (2009). Blinded by the light: the growing complexity of p53. Cell..

